# Demonstration of T-Cell Monotypia Using Anti-TCRbeta1/2 (*TRBC1*/2) Immunostaining as a Rapid and Cost-Effective Alternative to PCR-Based Clonality Studies for the Diagnosis of T-Cell Lymphoma

**DOI:** 10.3390/diagnostics14222479

**Published:** 2024-11-06

**Authors:** Elizabeth J. Soilleux, Daniel T. Rodgers, Jinlong J. Situ, Shelley C. Evans, Venkata N. Konda, Han-Chieh Yang, Jianxiong Pang, Isabella Gilbey Smith, Pete Rajesh, Maryam Salimi, Soo Weei Ng, Julia Jones, Jodi L. Miller, Rachel Etherington, Margaret Ashton-Key, Graham Ogg

**Affiliations:** 1 Department of Pathology, University of Cambridge, Cambridge CB2 0SP, UK; jjs76@cam.ac.uk (J.J.S.); vnk24@cam.ac.uk (V.N.K.); hcy23@cam.ac.uk (H.-C.Y.); jianxiong.pang@crick.ac.uk (J.P.); isabellagilbey@gmail.com (I.G.S.); pr506@cam.ac.uk (P.R.); 2 Haematopathology and Oncology Diagnostic Service (HODS), Cambridge University Hospitals NHS Foundation Trust, Cambridge CB2 0QQ, UK; 3 Human Research Tissue Bank, Cambridge University Hospitals NHS Foundation Trust, Cambridge CB2 0QQ, UK; daniel.rodgers@nhs.net; 4 MRC Translational Immune Discovery Unit, MRC Weatherall Institute of Molecular Medicine, University of Oxford, Oxford OX3 9DS, UK; maryam.salimi1@nhs.net (M.S.); jessica.ng@rdm.ox.ac.uk (S.W.N.); graham.ogg@ndm.ox.ac.uk (G.O.); 5 Cancer Research UK Cambridge Institute, Li Ka Shing Centre, University of Cambridge, Cambridge CB2 0RE, UK; julia.jones@cruk.cam.ac.uk (J.J.); jodi.miller@cruk.cam.ac.uk (J.L.M.); 6 Department of Cellular Pathology, University Hospital Southampton, Southampton SO16 6YD, UK; meg.ashton-key@soton.ac.uk

**Keywords:** lymphoma, monotypia, clonality, immunohistochemistry, diagnostic test, formalin-fixed paraffin-embedded tissue

## Abstract

Background/Objectives: T-cell lymphomas are often histologically indistinguishable from benign T-cell infiltrates, and diagnosis typically relies on slow, complex, and expensive multiplexed PCR reactions, requiring significant training and experience to interpret them. We aimed to raise highly specific antibodies against the two alternatively used and very similar T-cell receptor beta constant regions, TCRbeta1 and TCRbeta2, encoded by the *TRBC1* and *TRBC2* gene segments, respectively. We sought to demonstrate the feasibility of detecting TCRbeta1 and TCRbeta2 immunohistochemically in routine clinical (formalin-fixed, paraffin-embedded (FFPE)) tissue sections as a novel diagnostic strategy for T-cell lymphomas. Methods: Recombinant rabbit antibodies were validated using Western blotting and FFPE immunostaining of T-cell leukemia lines. The immunostaining of FFPE tissue containing benign and lymphomatous T-cell populations was undertaken, with corroboration by BaseScope^TM^ high-sensitivity in situ hybridization and quantitative real-time PCR (Q-PCR). An additional Q-PCR literature review and analysis of publicly available RNAseq data was used to determine the TCRbeta2/TCRbeta1 ratio cut-off to separate benign and malignant T-cell populations. Results: Our TCRbeta1/TCRbeta2 antibody pair gave highly specific FFPE tissue staining. All benign samples analyzed (immunohistochemically, by BaseScope^TM^, by Q-PCR, and by RNAseq data analysis) had TCRbeta1/TCRbeta2 or *TRBC1/TRBC2* ranges well within the previously published flow cytometric benign range (TCRbeta2/TCRbeta1 = 0.18:1–5.7:1), while samples of T-cell lymphoma did not. One out of thirteen (7.7%) lymphoma samples showed some detectable TCRbeta1/TCRbeta2 protein co-expression, and 4 out of 13 (30.8%) T-cell lymphomas showed a *TRBC1/TRBC2* transcript co-expression using BaseScope^TM^. Conclusions: Analyzing T-cell monotypia immunohistochemically, analogous to B-cell monotypia (kappa: lambda ratio for B-cell and plasma cell neoplasms), could make the diagnosis of T-cell lymphomas cheaper, quicker, and more accurate. Larger studies are needed to validate our antibodies for clinical use.

## 1. Introduction

The aggressive malignancy, T-cell lymphoma (TCL), has a global annual incidence of 60,000 and rising, and it comprises 10–15% of all lymphomas. TCL can cause masses, fevers, sweats, weight loss, skin rashes, anemia, bleeding, opportunistic infections, and ultimately death [1]. TCL is difficult, expensive, and time-consuming to diagnose, with patients often requiring multiple biopsies over a period of months or years due to initial biopsies giving inconclusive results. For skin TCL, the diagnostic delay (first symptoms to diagnosis) was a median of 36 months in one study [2] and 2–10 years in another [3]. Angioimmunoblastic TCL, with a 33% 5-year survival, is one of the more common systemic TCLs [4]. In total, 28/77 (36%) patients in one study had their first biopsy misdiagnosed, requiring repeat biopsy, with the median time between the misdiagnosis and the final diagnosis of angioimmunoblastic TCL being 2.3 [0.4 to 29] months [5]. The effects of diagnostic delay are best appreciated in enteropathy-associated T-cell lymphoma (EATL), with a median survival of 7 months [6]. EATL develops in the small intestine of up to 50% of patients with refractory celiac disease (RCD). RCD, essentially a low-grade intraepithelial TCL [6], occurs in celiac disease (CD; gluten-sensitivity) and is defined by a lack of clinical and histological response to a strict gluten-free diet. RCD is very difficult to distinguish from uncomplicated CD on a small intestinal biopsy, requiring repeated endoscopic biopsies, incurring a median diagnostic delay of 27 (4–58) months [7]. Timely and accurate RCD diagnosis with earlier intervention, before full-blown EATL, would substantially improve patient outcomes.

For T-cell lymphomas, composed of small-to-medium-sized cells (e.g., peripheral T-cell lymphoma, angioimmunoblastic lymphoma, and mycosis fungoides [1]), their morphological and immunophenotypic distinction from benign lymphocytic infiltrates can be very challenging. Diagnosis often necessitates multiplexed PCR T-cell clonality studies performed on DNA extracted from tissue, followed by the skilled interpretation of the resulting fragment analysis [8]. Current clonality studies lack an architectural and morphological context and are only undertaken in large specialist centers. The need to wait for the results of clonality studies can delay diagnosis by 1–2 weeks or more [8].

In comparison, a much smaller proportion of suspected B-cell lymphomas requires clonality studies because B cells show immunoglobulin light chain restriction, permitting a comparison of the numbers of cells expressing kappa and lambda light chains by either immunohistochemistry or in situ hybridization to look for monotypia. If all the cells in a B cell or plasma cell population express light chains of one type, that population of cells is said to be monotypic for the light chain and is likely to represent a lymphoma or plasma cell neoplasm. On the other hand, if the cells in a B cell or plasma cell population express a mixture of light chains of both types, that population of cells is said to be polytypic for the light chain and is likely to be benign [9,10,11].

**Figure 1:**
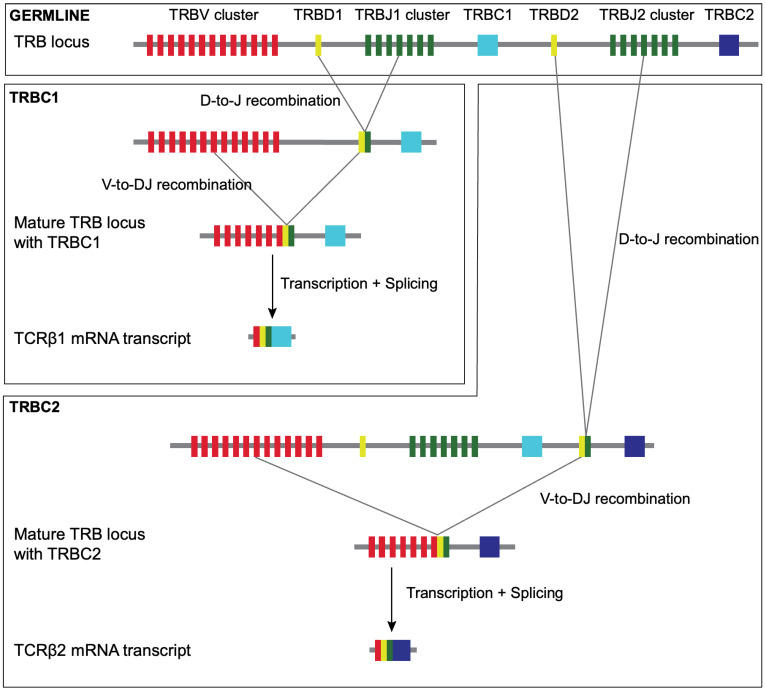
*TRB* gene rearrangement: the process of *TRB* gene rearrangement generates mutually exclusive *TRBC1* and *TRBC2* transcripts. The germline, unrearranged *TRB* locus consists of one V-gene cluster (*TRBV* cluster) followed by two ‘D-J-C’ gene clusters (*TRBD1-TRBJ1-TRBC1* and *TRBD2-TRBJ2-TRBC2*). During the recombination of the *TRB* locus in T-cell development, only one of the ‘D-J-C’ gene clusters is used to form the rearranged *TRB* locus. The *TRB* rearrangement occurs in a specific order, with the D-to-J recombination occurring first. This either joins a *TRBD1* gene segment with a *TRBJ1* gene segment or joins a *TRBD2* gene segment with a *TRBJ2* gene segment. V-to-DJ recombination occurs next, with a *TRBV* gene segment being brought adjacent to the recombined DJ segments to form a rearranged *TRB* locus. If productive VDJ recombination incorporates D- and J-gene segments originating from *TRBD1* and *TRBJ1* clusters, the rearranged *TRB* locus will contain *TRBC1*, which will encode the constant region of the T-cell receptor (TCR) beta. If the D- and J-gene segments originate from *TRBD2* and *TRBJ2* clusters, this will excise *TRBC1,* and the constant region of TCRbeta will instead be encoded by *TRBC2* [12,13,14].

The germline *TRB* locus (Figure 1) consists of one V-gene segment cluster followed by two ‘D-J-C’ gene clusters (*TRBD1-TRBJ1-TRBC1* and *TRBD2-TRBJ2-TRBC2*). During V-D-J-C segment recombination in T-cell development, only one of the ‘D-J-C’ gene clusters is used. Each mature alpha–beta T-cell, therefore, expresses a functionally rearranged T-cell receptor (TCR) that is either *TRBC1* or *TRBC2* in type, giving TCRbeta1 or TCRbeta2 protein expressions, respectively (Figure 1) [12,13,14]. In principle, this provides the opportunity to detect TCRbeta1 or TCRbeta2 protein expressions in a manner analogous to kappa and lambda light chain detection in B cells.

The TCRbeta1 and TCRbeta2 constant region amino acid sequences are >97% identical [14]. TCRbeta1 and pan-TCRbeta detection has been used successfully for flow cytometry for TCL diagnosis in blood and bone marrow samples [15], and an anti-TCRbeta2 antibody was recently produced by rational design [16]. An anti-TCRbeta1 antibody has also been used on frozen sections of tissue, comparing results with a pan-TCRbeta antibody [17]. While this TCRbeta1/TCRbeta2 antibody pair does not work on standard clinical FFPE biopsy material [16,17], an anti-TCRbeta1 and a pan-TCRbeta antibody were recently used on FFPE tissue for the diagnosis of cutaneous T-cell lymphomas [18].

Despite the similarity between the two TCRbeta-1 and TCRbeta-2 proteins, we successfully generated pairs of isotype-specific monoclonal antibodies, which work on routine clinical (formalin-fixed paraffin-embedded (FFPE)) material, which we validated on cell lines and benign and lymphomatous FFPE tissue, corroborating the TCRbeta 1:2 ratio by quantitative real-time (Q-PCR) for the *TRBC1*/*TRBC2* transcripts. Additionally, we analyzed RNAseq data and undertook real-time PCR on additional tissue samples to demonstrate that populations of benign polyclonal T-cells in blood and tissue have a *TRBC1*/*TRBC2* ratio close to 1:1, which is a more favorable ratio than kappa/lambda, making the skewing of this ratio readily detectable both in immortalized T-cell lines and in T-cell lymphomas. This antibody pair has the potential to revolutionize the diagnosis of T-cell lymphoma.

## 2. Materials and Methods

### 2.1. Production of Recombinant Rabbit Antibodies

**Figure 2:**
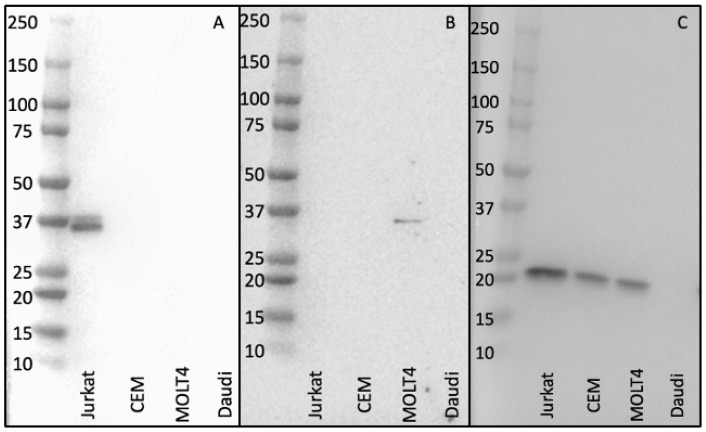
Western blotting demonstrated the specificity of antibody supernatants ROX7 (**panel A**) and ROX11 (**panel B**) of lysates from cell lines such as Jurkat (TCRbeta1), CEM (low expression level of TCRbeta2), MOLT4 (high expression level of TCRbeta2), and Daudi (B cell line; negative for TCRbeta1 and TCRbeta2). ROX7 gave a 37 kD band with Jurkat (TCRbeta1-expressing cells) but none of the other cell types, while ROX11 gave a 37 kD band with MOLT4 (TCRbeta2-expressing cells), but none of the other cell types, with the TCRbeta2 protein level in CEM cells presumably being below the limit of detection. Western blotting control was undertaken with a rabbit polyclonal anti-CD3 antibody, directed against the 20 kD CD3ε (epsilon) chain [19], giving a band of an appropriate size with the three T-cell lines, but not with the Daudi B-cell line (**panel C**).

**Figure 3:**
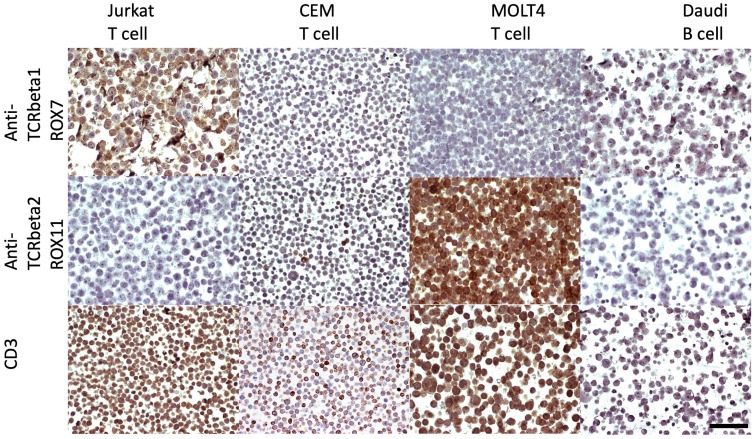
FFPE cell pellets of Jurkat (TCRbeta1-expressing cell line), CEM (low-level TCRbeta2-expressing cell line), MOLT4 (high-level TCRbeta2-expressing cell line), and Daudi (B cell) lines stained with antibodies ROX7 (TCRbeta1-specific), ROX11 (TCRbeta2-specific), and polyclonal rabbit anti-CD3, detected using anti-rabbit secondary antibody, the horseradish peroxidase (HRP) system, and diaminobenzidine DAB) to give brown positive staining. Hematoxylin nuclear counterstaining is blue-purple. BaseScope^TM^ corroboration is included in Supplementary Figure S3. Q-PCR data provided further corroboration, with *TRBC2*:*TRBC1* ratios as follows: Jurkat: *TRBC1*/*TRBC2* = 11.43:1, CEM: *TRBC2*/*TRBC1* = 14.86:1, MOLT4: *TRBC2*/*TRBC1* = 2.20 × 10^6^:1, and Daudi: neither transcript detectable. Further details of the Q-PCR are included in Supplementary Figures S1 and S2. Scale bar (bottom right-hand panel) pertains to all panels and is 50 microns.

Rabbits were immunized with TCRbeta1- or TCRbeta2-derived peptides in order to produce TCRbeta1-specific (ROX7) and TCRbeta2-specific (ROX11) recombinant rabbit IgG antibodies. Briefly, peptides were synthesized chemically and corresponded to the extracellular region of difference between TRBC1 and TRBC2, including “EDLNKVF” versus “EDLKNVF”, and conjugated to KLH. The specificity of the antibodies binding to the peptide variants was determined by Western blotting and the immunostaining of immortal cell lines as described (Figure 2 and Figure 3), with further confirmation using primary samples and concomitant quantitative real-time reverse transcription PCR (Q-PCR) and in situ hybridization (Table 1). Additional rabbit antibody was produced using recombinant technology via their expression in HEK293T cells. Antibodies were purified by chromatography, using size-exclusion HPLC (Superdex 200, GE28-9909-44, Sigma Aldrich, Gillingham, UK), and were confirmed to be of >98% purity.

### 2.2. Cell Culture and Production of FFPE Cell Line Pellets

Jurkat (TCRbeta1-expressing), CEM (TCRbeta2-expressing), MOLT-4 (TCRbeta2-expressing), and Daudi (B-cell; negative for TCBRbeta1 and TCRbeta2) immortal lymphoid lines were cultured [20] and either used for Western blotting or to produce FFPE cell pellets. For cell pellet production, following centrifugation, cytological pellets were processed to FFPE material. Briefly, human plasma (obtained from the National Health Service Blood Transfusion Service, Cambridge) was added to the cell pellet, followed by 2 drops of bovine thrombin at 50 units/mL (BTUB291, Diagnostic Reagents Ltd, Thame, UK) in order to form a cell pellet clot. Pellet clots were labeled with different colored inks to indicate their identity and were fixed in neutral buffered formalin for 24 h, then processed to paraffin. Three micron sections of the cell pellets were cut onto charged microscope slides and used for immunostaining. Two TCRbeta2-expressing cell lines were used, as Q-PCR indicated that CEM and MOLT-4 differed in their expression levels of *TRBC* transcripts.

### 2.3. Western Blotting

Western blotting was performed under standard reducing conditions. Briefly, cell lysates were obtained using RIPA Lysis Buffer (50 nM Tris, 0.1% SDS, 150 mM NaCl, 0.5% DOC, 1% Nonidet P40) plus protease inhibitors (cOmplete, Mini, EDTA-free Protease Inhibitor Cocktail, Roche, Mannheim, Germany, 11836170001 made in distilled water to a 100× stock). Total protein concentrations were measured using the Pierce BCA Protein Assay Kit (Thermo Fisher Scientific, Bishop’s Stortford, UK). Equal amounts of total proteins (25 μg per lane) were resolved by SDS-PAGE and transferred to the Invitrolon PVDF membrane (Invitrogen LC2005, Carlsbad, CA, USA) and blocked with 5% skimmed milk/TBST. For protein detection, the following antibodies, diluted in 5% skimmed milk/TBST, were used: the rabbit IgG monoclonal anti-TCRbeta1 (ROX7, at 0.16 ng/μL), rabbit IgG monoclonal anti-TCRbeta2 (ROX11 at 0.32 ng/μL), and rabbit polyclonal anti-CD3 (Dako Omnis, ready-to-use preparation, diluted 1/10, Agilent, Santa Clara, CA, USA). After incubation with appropriate HRP-conjugated secondary anti-rabbit antibodies (Jackson Immuno Research Europe, Cambridge, UK) and the enhanced chemiluminescence (ECL) reagent (Promega Corporation, Madison, WI, USA), Western blotting bands were detected by the ChemiDoc Touch Imaging System (Bio-Rad, Hercules, CA, USA).

### 2.4. FFPE Clinical Tissue Samples

Histological patient material (lymphomas and benign material, Table 1 and Table 5) was obtained from the Cambridge University Hospitals NHS Foundation Trust Human Research Tissue Bank (HTRB) or from the Department of Cellular Pathology, University Hospital Southampton NHS Foundation Trust, Southampton, UK, with full ethical approval (IRAS: 162057; PI: Professor E. Soilleux).

### 2.5. Immunostaining

The immunostaining of cell pellets and tissue sections with rabbit IgG monoclonals anti-TCRbeta1 (ROX7) and anti-TCRbeta2 (ROX11) was performed on a Leica Bond-III platform, with 20 min heat pre-treatment in an acid buffer, Epitope Retrieval Solution 1 (Cat No. AR9961, Leica Biosystems, Newcastle-upon-Tyne, UK), in conjunction with the Polymer Refine Detection System Cat No. DS9800, Leica Biosystems, Newcastle-upon-Tyne, UK).

### 2.6. BaseScope^TM^ RNA In Situ Hybridization

The detection of TRBC1/TRBC2 targets, as well as positive and negative control targets, was performed on FFPE cell pellets and tissue sections using the Advanced Cell Diagnostics (ACD) BaseScope™ LS Reagent Kit (Cat. No: 323600) and various BaseScope LS probes (BA-hSequexBS1-3zz-st-C1 and BA-hSequexBS2-3zz-st-C1, cat. Nos 1139338-C1 and 1139338-C1, respectively, designed against the 3′ UTRs of TRBC1 and TRBC2 (Genbank accession numbers: M12887.1 and M12888.1)), together with PPIB (Genbank: NP_000933.1; peptidylprolyl isomerase B)-positive and DapB (Genbank: EF191515.1)-negative control probes (BA-Hs-PPIB-3zz (cat. No. 70-10-38) and BA-DapB-3zz (cat. No. 70-10-18), respectively (ACD, Hayward, CA, USA)) [21]. Briefly, sections were cut at 3 um and baked for 1 h at 60 °C before loading onto a Bond RX instrument (Leica Biosystems, Newcastle-upon-Tyne, UK). Slides were deparaffinized and rehydrated on the instrument before pre-treatments using Epitope Retrieval Solution 2 (Cat No. AR9640, Leica Biosystems, Newcastle-upon-Tyne, UK) at 95 °C for 15 min, and the ACD Enzyme from the LS Reagent kit at 40 °C for 15 min. Probe hybridization and signal amplification were performed according to the manufacturer’s instructions. The fast red detection of each target was performed on the Bond Rx using the Bond Polymer Refine Red Detection Kit (Leica Biosystems, Newcastle-upon-Tyne, UK, Cat. No. DS9390) according to the ACDs protocol. The slides were then removed from the Bond Rx and were heated at 60 °C for 1 h, dipped in Xylene, and mounted using the EcoMount Mounting Medium (Biocare Medical, Concord, CA, USA, Cat No. EM897L). The slides were imaged on the Aperio AT2 (Leica Biosystems, Newcastle-upon-Tyne, UK) to create whole slide images. Images were captured at 40× magnification, with a resolution of 0.25 microns per pixel.

### 2.7. Analysis of Histological Results

Histological results were analyzed by an experienced consultant hematopathologist (EJS) using an Olympus BX53 microscope (Olympus, Tokyo, Japan). The photography of stained slides was undertaken with a Lumenera Infinity 2 camera (Lumenera, Ottawa, ON, Canada) mounted on an Olympus BX53 microscope.

### 2.8. Quantitative Real-Time Reverse Transcription PCR

RNA was extracted from FFPE tissue using the RNeasy^®^FFPE Kit (Qiagen, Manchester, UK), and cDNA was synthesized using the SuperScript^®^ III First-Strand Synthesis System (Life Technologies, Paisley, UK) according to the manufacturer’s instructions. Seven fresh frozen tissue samples were also obtained from HTRB, with full ethical approval (IRAS: 162057; PI: Professor E. Soilleux), and RNA was extracted using the RNeasy^®^ Plus Mini kit (Qiagen), as per the manufacturer’s instructions, with cDNA synthesis as above. Real-time PCR reactions were performed using the Power SYBR^®^ Green PCR Master Mix (Applied Biosystems, Warrington, UK) in a Quantstudio 6 (Thermo Fisher Scientific, Bishop’s Stortford, UK) with the following primer sets: *TRBC1*-forward CTTGTGTTGATGGCCATGGT, *TRBC1*-reverse. AGCGCTGGCAAAAGAAGAATG; *TRBC2*-forward TGGTCAAGAGAAAGGATTCCAG, *TRBC2*-reverse AGGAACACAGATTGGGAGCA; PPIB-forward AGATGTAGGCCGGGTGATCT; and PPIB-reverse CTCCGCCCTGGATCATGAAG PCR in 10 µL mixtures containing 5 µL of iTaq SYBR^®^ Green Supermix, 0.5 µL of forward and reverse primers, 2 µL of the cDNA template and 2 µL of nuclease-free water. The following conditions were used: initial denaturation at 95 °C for 30 s, then 40 cycles of 95 °C for 15 sec, and 63 °C for 1 min. To confirm that identical amounts of the *TRBC1* and *TRBC2* template would give very similar Q-PCR results (i.e., to confirm relative PCR efficiencies were very similar), a construct was synthesized in the pUCIDT (KanR) vector ((Integrated DNA Technologies (IDT), Leuven, Belgium), for use as a PCR template (Thermo Fisher Scientific, Bishop’s Stortford, UK). The insert comprised the 3′UTR of *TRBC1*, then a 24-base random spacer sequence in which there was an ECOR1 site, followed by the 3′UTR of *TRBC2* (Supplementary Figures S1 and S2). For tissue samples, Sanger sequencing of a selection of amplicons was used to confirm amplicon specificity.

### 2.9. Bioinformatic Analysis of RNAseq Data

*TRB* RNAseq data were obtained from two sources: we used in-house data from our study of duodenal biopsies from 12 patients with celiac disease and 8 healthy donors [22]. Briefly, fluorescence-activated cell sorting (FACS) was performed to separate the lymphocytes into CD4+ and CD8+ T-cell subsets prior to sequencing, using the amplicon-rescued multiplex (ARM)-PCR (iRepertoire Inc., Huntsville, AL, USA). We also analyzed data from a study of peripheral blood CD4+ T-cells from 5 healthy donors who had undergone flow cytometric sorting into 8 T-cell subsets, following which bulk TCRbeta repertoire sequencing was undertaken using the Milaboratories system (Milaboratories, Sunnyvale, CA, USA) [23]. We used MiXCR [24] to analyze the read count and the C-segment usage of each unique clonotype. We thus calculated (a) the mRNA transcript *TRBC1*/*TRBC2* ratio and (b) the ratio of *TRBC1*-restricted cells/*TRBC2*-restricted cells. For the latter ratio, given the huge diversity of the TCR repertoire [13], the assumption was made that each unique TCR sequence in the sample was likely to represent a single T-cell, and thus, the read count for each unique clonotype was normalized to 1.

## 3. Results

### 3.1. Validating TCRbeta1 and TCRbeta2-Specific Antibodies Applicable to Formalin-Fixed Paraffin-Embedded (FFPE; Routine Clinical) Tissue

#### 3.1.1. Western Blotting

Western blotting demonstrated the specificity of antibody supernatants ROX7 (Figure 2A) and ROX11 (Figure 2B) of lysates from the cell lines Jurkat (TCRbeta1), CEM (low expression level of TCRbeta2), MOLT4 (high expression level of TCRbeta2), and the Daudi (B cell line; negative for TCRbeta1 and TCRbeta2). Control Western blotting was undertaken with a rabbit polyclonal anti-CD3 antibody directed against the 20 kD CD3ε (epsilon) chain [19] (Figure 2C). The presence of a second faint band, with a slightly higher molecular weight, in the Jurkat cell lysate, is likely because TCRbeta can exist in differential glycosylation states [25,26,27,28,29].

#### 3.1.2. Immunostaining of FFPE Cell Line Pellets

The FFPE cell pellets of Jurkat (TCRbeta1), CEM (low level of TCRbeta2), MOLT4 (high level of TCRbeta2), and Daudi (B cell) lines were stained with antibodies ROX7 (TCRbeta1-specific) and ROX11 (TCRbeta2-specific), with a rabbit polyclonal CD3 antibody as a control. This demonstrated that ROX7 and ROX11 gave highly specific staining for TCRbeta1 and TCRbeta2, respectively, on FFPE tissue (Figure 3), commensurate with corresponding BaseScope^TM^ data for the *TRBC2* and *TRBC1* transcripts (Supplementary Figure S3).

### 3.2. Immunohistochemical Expression Pattern and TCRbeta2/TCRbeta1 Ratios in FFPE (Routine Clinical) Tissue Samples Containing Populations of Benign T-Cells

**Figure 4:**
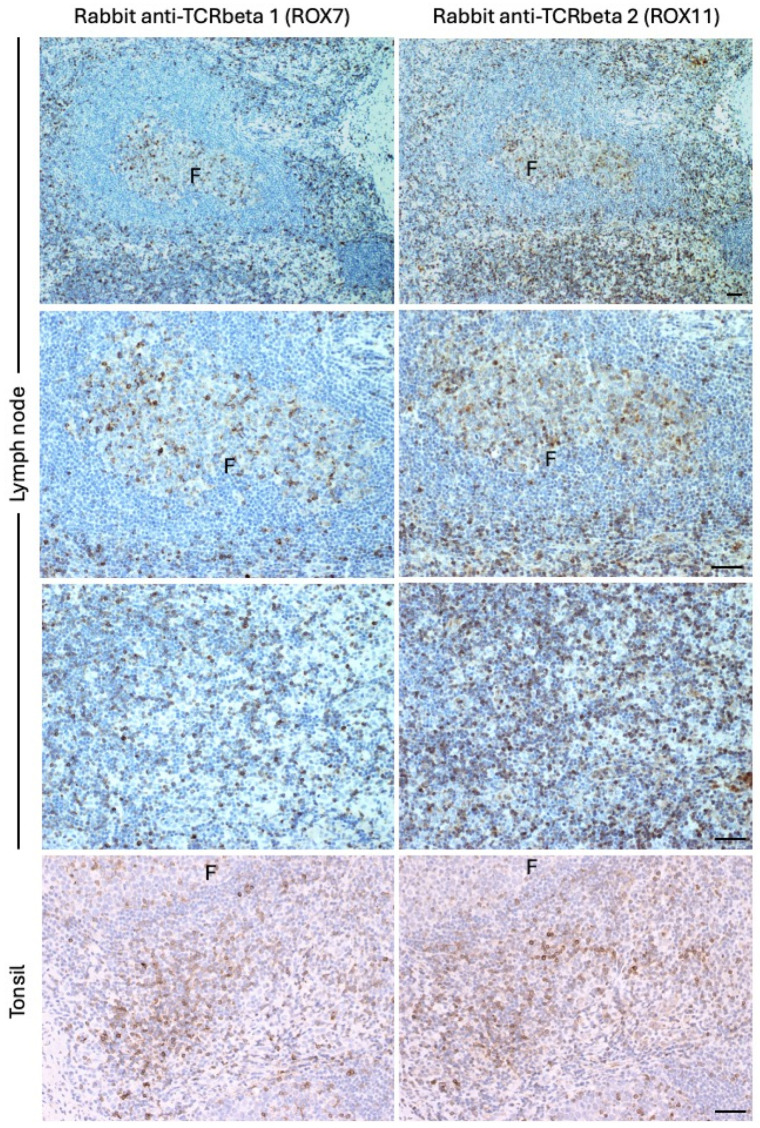
Photomicrographs of the FFPE lymph node (case 3; upper 6 panels) and tonsils (case 1; lower 2 panels) immunostained with ROX7 (anti-TCRbeta1; left-hand panels) and ROX11 (anti-TCRbeta2; right-hand panels). Positive cells appear brown. Roughly equal numbers of T-cells are positive with each antibody, and these are either T-follicular helper cells located in the centers of B-cell follicles (follicles labeled F) or T-cells present in the lymph node paracortex (third panel from top) or T-zone of the tonsils (lowest panels). Scale bars of the right-hand images pertain to each pair of images and represent 50 microns. BaseScope^TM^ corroboration is included in Supplementary Figure S4, with corresponding Q-PCR data in Table 1.

We then applied the ROX7 and ROX 11 antibodies (TCRbeta1 and TCRbeta2-specific antibodies, respectively) to FFPE tissues containing benign populations of T-cells (Figure 4, Table 1) and observed that very close to equal numbers of T-cells were positive for TCRbeta1 and TCRbeta2. The ratio of TCRbeta2-expressing to TCRbeta1-expressing T-cells ranged between 0.67:1 and 1.5:1. This was closely corroborated by the *TRBC2*/*TRBC1* BaseScope^TM^ ratios, which ranged between 1:1 and 2.3:1 (Table 1, Supplementary Figure S4). In addition, Q-PCR for the *TRBC1* and *TRBC2* transcripts corroborated the immunostaining results by giving a *TRBC2*:*TRBC1* ratio of between 0.73:1 and 2.82:1 (Table 1). In terms of spatial localization, T-cells in all physiological T-cell compartments in the lymph nodes and tonsils expressed either TCRbeta1 or TCRbeta 2 (Figure 4), commensurate with corresponding BaseScope^TM^ data for the *TRBC2* and *TRBC1* transcripts (Supplementary Figure S4).

#### 3.2.1. Corroboration by Quantitative Real-Time Reverse Transcription PCR (Q-PCR)

The TCRbeta2+/TCRbeta1+ cell ratios in benign FFPE (routine clinical) tissue samples were corroborated by means of Q-PCR (Table 1) and gave *TRBC2*/*TRBC1* transcript ratios between 0.73:1 and 2.82:1. In addition, seven fresh frozen tissue samples were obtained and gave *TRBC2*/*TRBC1* transcript ratios between 1.72:1 and 4.01:1 (Table 2). While these Q-PCR transcript ratios are an indication of the likely ratios of TCRbeta2+/TCRbeta1+ cell numbers, the results may be confounded by cell-to-cell variation in expression levels, underlining the advantages of using tissue sections with associated architecture. Further details of our Q-PCR strategy are included in Supplementary Figures S1 and S2.

#### 3.2.2. Analysis of TRBC2/TRBC1 Transcript Ratios in Publicly Available Datasets

In order to understand the optimal TCRbeta2+/TCRbeta1+ ratio at the cellular level that could provide a safe cut-off between benign and malignant T-cell populations in clinical practice, we set out to determine the physiological *TRBC2/TRBC1* transcript ratio, analyzing a publicly available CD4+ T-cell RNAseq dataset (Table 3; Supplementary Table S1) [23] and an in-house dataset of duodenal T-cells from celiac disease patients and healthy donors [22]. Details of cell-sorting strategies are included in the original publications of these datasets [22,23].

In most samples, particularly the CD8+ subsets, there were slightly more *TRBC2* than *TRBC1* transcripts, which is in keeping with our Q-PCR results (Table 1 and Table 2) and published data [16,30,31,32,33].

We recognized the need to assess the likely ratio of *TRBC2*-expressing cells to *TRBC1*-expressing cells as a better correlation of what might be seen histologically. *TRBC* expression levels may vary from one T-cell to another. Because peripheral blood T-cell populations are highly diverse [13] and, even in strong immune responses, no more than 1% of T-cells show an identical clonotype [8], we assumed that each TCR clonotype was present in only one cell, allowing for the calculation of the likely *TRBC2*+ cell/*TRBC1*+ cell ratio (Table 3).

#### 3.2.3. Collation of TCRbeta2/TCRbeta1 Ratios in Published Data

Published data from flow cytometry studies using a TCRbeta1 and a pan-TCRbeta antibody provide an estimate of the likely range of TCRbeta2+/TCRbeta1+ cell ratios in both peripheral blood and certain tissues and body fluids [30,31,32,33]. These results provide a further surrogate for a TCRbeta2+/TCRbeta1+ cell ratio. One recent publication paired a rationally designed anti-TCRbeta2 antibody with an anti-TCRbeta1 antibody for flow cytometry. Collectively, these results demonstrate how designating samples with a TCRbeta2/TCRbeta1 ratio outside the range of 0.18:1–5.7:1 could be taken to indicate T-cell lymphoma (Table 4) [16]. However, a narrower range might be used if additional data, such as morphology and architecture, can be overlain.

#### 3.2.4. Calculation of a Pragmatic Cut-Off for TCRbeta2/TCRbeta1 Ratios

All our analyses of benign T-cell populations (Table 1, Table 2 and Table 3) fall closer to a 1:1 ratio than the ratios in the published data that we collated (Table 4). This indicates that, in solid tissue samples, it is likely to be clinically safe to conclude that a TCRbeta2/TCRbeta 1 ratio falling outside the range of 0.18:1–5.7:1 indicates a high likelihood of T-cell lymphoma [16]. It may be possible to narrow this ratio following larger studies, which should increase the sensitivity of the detection of T-cell lymphoma.

### 3.3. Application of TCRbeta1 and TCRbeta2-Specific Antibodies to FFPE Tissue Samples of T-Cell Lymphoma

**Figure 5:**
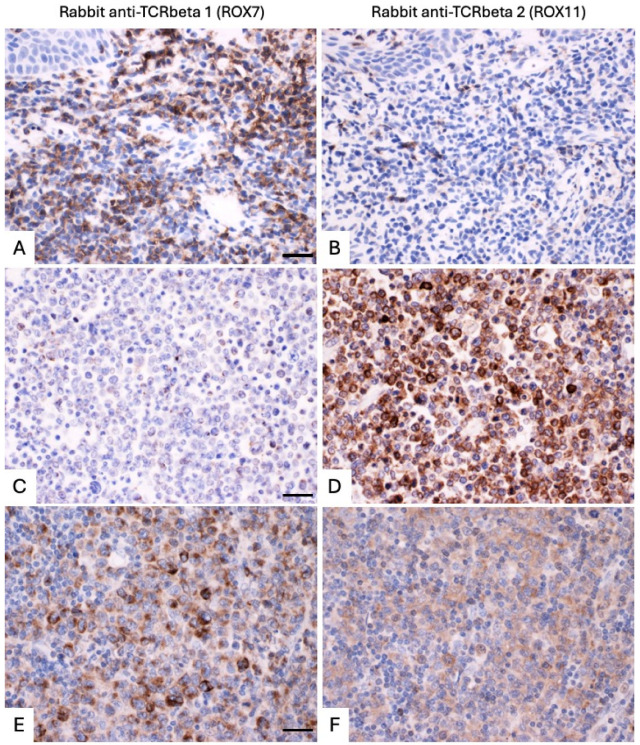
Photomicrographs of FFPE sections of T-cell lymphomas immunostained with ROX7 (anti-TCRbeta1, left-hand panels) and ROX11 (anti-TCRbeta2, right-hand panels). (**A**,**B**). Cutaneous lymphoma (transformed mycosis fungoides) in scrotal skin (case 16) in (**A**,**B**) showing clear TCRbeta1-restriction. (**C**,**D**). Peripheral T-cell lymphoma, NOS, in a lymph node (case 11), showing clear, TCRbeta2-restriction. (**E**,**F**). Peripheral T-cell lymphoma, NOS, in a lymph node (case 9), showing clear well-defined membranous TCRbeta1 expression, with some weaker cytoplasmic TCRbeta2 co-expressions. All results were corroborated by Q-PCR (Table 5) and BaseScope^TM^ (Supplementary Figure S5). Scale bars (left-hand panels) are 20 microns and pertain to paired left and right-hand panels.

**Figure 6:**
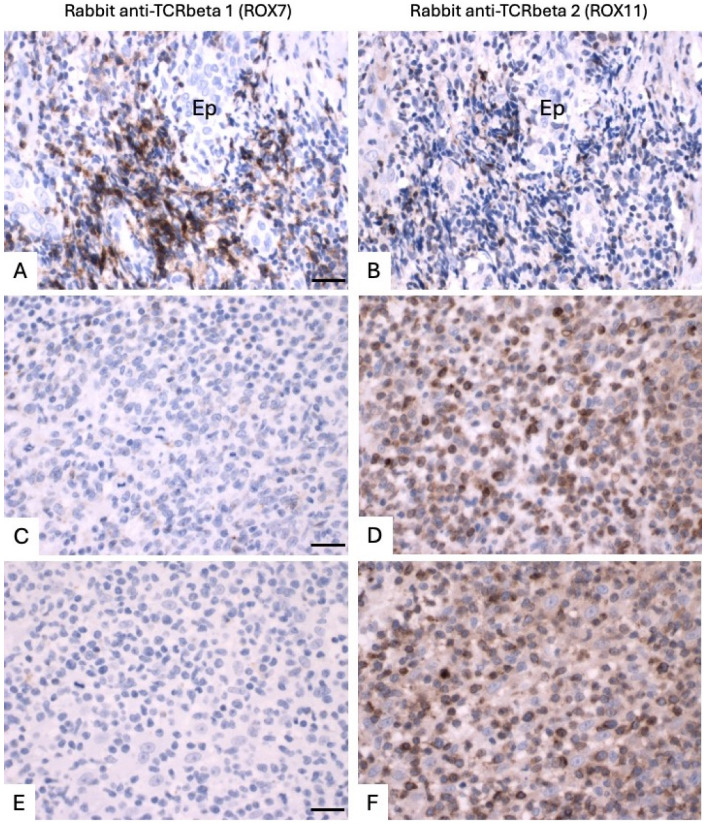
Photomicrographs of FFPE sections of T-cell lymphomas immunostained with ROX7 (anti-TCRbeta1, left-hand panels) and ROX11 (anti-TCRbeta2, right-hand panels). (**A**,**B**). Unclassifiable CD4+ cutaneous T-cell lymphoma (case 13) showing TCRbeta1-restriction. (**C**,**D**). Cutaneous T-cell lymphoma (transformed mycosis fungoides) (case 17) showing TCRbeta2 restriction, although at the transcript level, there is cytoplasmic *TRBC2*, as seen in all the other cases examined, but very strong nuclear *TRBC1* (Supplementary Figure S6, panels C and D). (**E**,**F**). CD8-positive cutaneous T-cell lymphoma, possibly acral lymphoma (case 21), showing clear TCRbeta2-restriction. Scale bars (left-hand panels) are 20 microns and pertain to paired left and right-hand panels. BaseScope^TM^ corroboration is included in Supplementary Figure S6, with Q-PCR data in Table 5. Ep, epidermis.

We then applied the ROX7 and ROX 11 (TCRbeta1 and TCRbeta2-specific) antibodies to a selection of FFPE tissues containing T-cell lymphomas (Figure 5 and Figure 6, Table 5). As with benign samples, high-quality staining was observed. Of the 13 lymphoma cases, 9 showed TCRbeta2-restriction, 3 showed TCRbeta1-restriction, and 1 showed dual expression, with strong membranous staining for TCRbeta1 and weaker cytoplasmic staining for TRCbeta2 (case 9, Figure 5 panels E,F). The direction of skewing was corroborated in all cases by the Q-PCR results (Table 5). Of the three TCRbeta1-restricted cases, two were *TRBC1*-restricted at the transcript level, with one showing dual transcript expression but with a *TRBC1* preponderance (case 19). Of the nine TCRbeta2-restricted cases, seven were *TRBC2*-restricted at the transcript level, and two showed dual transcript expression, with one of them (case 15) showing dual cytoplasmic transcript expression. The other (case 17) had a very unusual pattern of transcript expression, with cytoplasmic *TRBC2*, as seen in all the other cases examined, but very strong nuclear *TRBC1*. Given the lack of associated TCRbeta1 protein expression, this raises the possibility of translocation or mutation causing inappropriate transcript localization and precluding protein expression. The determination of the exact genomic basis of this observation was beyond the scope of this study.

Monotypia can be regarded as a surrogate for monoclonality, and, in this study, all the neoplastic T-cell populations showed either a TCRbeta1/TCRbeta2 or TCRbeta2/TCRbeta1 immunohistochemical ratio of at least 10:1, indicating that a TCRbeta2/TCRbeta1 ratio cut-off of 0.18:1–5.7:1 for suspected T-cell lymphoma could be used in clinical practice for solid tissue samples. In addition, one case co-expressed TCRbeta1 and TCRbeta2 at the protein level, but because all the cells showed the same patterns of expression, they could also be defined as monotypic. The interpretation of TCRbeta1/TCRbeta2 immunohistochemistry was significantly easier than the interpretation of the *TRBC1*/*TRBC2* BaseScope^TM^, as, with the latter, determining which nucleus dots of staining were associated with what was challenging, morphology was less well preserved due to cell pre-treatment methods, and there was more dual expression at the transcript than protein level. In summary, it would have been possible to determine, on the basis of TCRbeta1 and TCRbeta2 immunohistochemistry alone, that the T-cells were monotypic in all 13 lymphoma cases in this study, while neither BaseScope^TM^ nor Q-PCR could have given a conclusive result about monotypia in all cases, although both results corroborated the direction of TCR expression skewing in all cases.

## 4. Discussion

Here, we present a novel immunohistochemical assay that can separate benign from malignant T-cell populations by determining whether they are monotypic or polytypic for the TCRbeta constant region in a manner analogous to the kappa/lambda assay for B cells and plasma cells. If it is to be adopted widely as a new diagnostic test for T-cell lymphoma, the fact that this assay works on FFPE tissue is critically important. An assay of this nature could be used in routine clinical practice to give definitive results after 4–6 h, expediting the determination of T-cell clonal status. Such an assay compares very favorably with current DNA-based clonality tests, which generally take several weeks, thus permitting the much more rapid and reliable diagnosis of T-cell lymphomas [8].

Our novel approach has the advantage that TCRbeta restriction (i.e., T-cell monotypia versus polytypia) can be assessed alongside tissue architecture, T-cell morphology, and T-cell immunophenotype, further facilitating diagnosis. This permits a pathologist, using serial sections, or perhaps double immunostaining, to identify a population of T-cells based on a combination of morphology and/or immunophenotype and/or localization in the tissue section and then to assess whether that population is monotypic or polytypic for the TCRbeta constant region. This potentially allows a pathologist to ignore any TCRbeta constant region expression caused by T-cells that they interpret as inflammatory or reactive. Screening out the results from benign T-cells is difficult with current DNA-based clonality testing unless a time-consuming microdissection is undertaken, which means that a monoclonal result may not be identifiable against the polyclonal background that is generated by benign T-cells. Compared with the current situation, in which patients often require multiple biopsies over a period of months or years because of inconclusive results [2,3,4,5,6], this approach could avoid patient anxiety and improve clinical outcomes, as well as provide cost savings for health services.

The kappa/lambda light chain ratio starts at a range from 2:1 to 4:1 [1]. This physiological ratio readily permits the assessment of skewing as an indicator of the presence of a light chain-restricted population in the majority of cases. The physiological TCRbeta2/TCRbeta1 ratio is more advantageous, at a very close 1:1 range, further facilitating the assessment of skewing. Future larger studies are needed to validate our findings and establish definitive cut-offs for clinical use. To establish these cut-offs, it will be necessary to investigate the potential for any skewing as a function of the site, age, or any specific non-neoplastic condition, including, for example, EBV-driven T-cell proliferation. In general, our work corroborates previously published results, indicating that a TCRbeta2/TCRbeta1 ratio skewed outside 0.18:1–5.7:1 might be regarded as corroborative of T-cell lymphoma in an appropriate clinicopathological context. Further studies may permit some narrowing of this range.

Although quantifying and comparing *TRBC1*/2 transcript levels by both the Q-PCR and BaseScope^TM^ was broadly corroborative of our TCRbeta1/2 immunohistochemical results, neither technique was as powerful in separating benign and malignant T-cell populations as immunohistochemical staining. Q-PCR is confounded by the number of transcripts per cell, as well as by the RNA derived from any benign lymphocyte populations in the pathological sample. Furthermore, the use of Q-PCR is unable to capitalize on the spatial and morphological context of the transcript expression, thus removing important diagnostic clues. A minor challenge of using BaseScope^TM^ was determining which dot (with each dot representing a transcript) should be ascribed to exactly which nucleus, but otherwise, it afforded many of the advantages of immunohistochemistry. While BaseScope^TM^ can helpfully assess the transcript ratio at the level of numbers of cells expressing *TRBC1* or *TRCB2*, interpretation may be difficult because of occasional dual *TRBC1*/*TRBC2* transcript expression. Dual *TRBC1*/*TRBC2* transcript expression appears to be more common in T-cell lymphomas (4/13 or 30.8% in our series) than kappa and lambda light chain co-expression in B-cell or plasma cell neoplasms [34,35,36]. The only published studies assessing dual *TRBC* transcript expression relate to benign T-cells and suggest that 1–7% express dual TCRbeta chains on the cell surface [37,38,39,40,41,42,43], and this level of dual expression would be too low to confound our TCRbeta1/2 immunohistochemical assay for T-cell lymphoma. Now that TCRbeta1 and TCRbeta2-specific antibodies are available, it will be possible to determine the proportion of benign T-cells with a dual TCRbeta1/2 expression in a range of tissues. It will also be possible to assess how frequently dual *TRBC*1/2 transcript expression leads to dual TCRbeta1/2 protein expression.

Overall, our data demonstrate two important aspects of assessing TCRbeta ratios in the diagnosis of T-cell lymphoma. Firstly, it is critical to assess the ratio of cells that are positive for each receptor, not simply the ratio of expressed transcripts. Secondly, to avoid the confounding effects of dual transcript expression, it is important to assess the receptors at the protein level rather than the transcript level. TCRbeta immunohistochemistry with our isotype-specific antibodies fulfills both of these points.

## 5. Conclusions

In summary, we describe the basis of a novel immunohistochemical assay for T-cell monotypia, which would make the diagnosis or exclusion of T-cell lymphoma more rapid, cost-effective, and accurate compared with current DNA-based clonality studies. It is applicable to standard clinical diagnostic workflows with FFPE tissue and has the potential for widespread adoption by haematopathologists and dermatopathologists.

## Figures and Tables

**Table 1:** Analysis of ratios of numbers of TCRbeta2/TCRbeta1-expressing cells in immunostained FFPE tissue sections containing benign T-cell populations, with corroboration by BaseScope^TM^ (Supplementary Figure S4) and Q-PCR (Table 1; Supplementary Figures S1 and S2).

Case Number	Site	Diagnosis	IHC Cell TCRbeta2/TCRbeta1	BaseScope^TM^ *TRBC2*:*TRBC1*	Q-PCR *TRBC2*:*TRBC1*
1	Tonsils	Benign, reactive	1.5:1	1.5:1	2.82:1
2	Lymph node	Benign, reactive	1.5:1	2.3:1	1.6:1
3	Lymph node	Benign, reactive	1.5:1	1.5:1	0.83:1
4	Lymph node	Benign, reactive	1.5:1	1.5:1	1.92:1
5	Skin (temple)	Lichenoid keratosis	1:1	1:1	1.76:1
6	Buccal mucosa	Lichen planus	1:1	1.5:1	0.98:1
7	Lymph node	T-cell/histiocyte-rich large B-cell lymphoma	0.67:1	1:1	0.73:1
8	Lymph node	Classic Hodgkin lymphoma	1.5:1	1.5:1	1.88:1

**Table 2:** *TRBC2/TRBC1* transcript ratios produced by Q-PCR on RNA extracted from fresh frozen tissue at the anatomical sites with the diagnoses shown.

Sample	Clinical Characterization	*TRBC2*/*TRBC1* Transcript Ratio (x:1)
TB15.0286	Tonsillitis	1.72:1
TB14.1744	Cholangiocarcinoma	2.33:1
TB15.0347	Recurrent tonsillitis	2.47:1
TB15.2568	Crohn’s disease	2.62:1
TB16.2261	Ulcerative colitis	3.41:1
TB17.1570	Inflamed skin	3.80:1
TB15.0750	Crohn’s disease	4.01:1

**Table 3:** In the dataset from Kasatskaya et al. [23], CD4+ T-lymphocytes derived from the peripheral blood of 5 healthy donors were sorted into 8 different phenotypes prior to undergoing T-cell receptor repertoire sequencing (Milaboratories, Sunnyvale, CA, USA). To produce our in-house dataset [22], duodenal T-lymphocytes from 12 celiac patients and 8 healthy controls were sorted into CD4+ and CD8+ subsets using fluorescence-activated cell sorting, prior to RNA extraction, followed by T-cell receptor repertoire sequencing (iRepertoire Inc., Huntsville, AL, USA). *TRBC2*/*TRBC1* ratios were calculated for each sample. The mean, range, and standard deviation of the ratios are stated for each group, and further details are included in Supplementary Table S1.

Publication Reference for Dataset Analyzed	Anatomical Site/Pathological Status	T-Cell Subset	Estimated Mean *TRBC2*-Expressing/*TRBC1*-Expressing Cell Ratio (and Range)	Standard Deviation
Kasatskaya et al. [23]	Peripheral blood	CD4+ T follicular helper	1.364 (range: 1.226 to 1.466)	0.094
		CD4+ T helper 1	1.381 (range: 1.249 to 1.569)	0.146
		CD4+ T helper 1-17	1.3 (range: 1.206 to 1.417)	0.086
		CD4+ T helper 17	1.435 (range: 1.288 to 1.606)	0.16
		CD4+ T helper 2	1.344 (range: 1.244 to 1.436)	0.082
		CD4+ T helper 22	1.264 (range: 1.204 to 1.362)	0.059
		CD4+ non-classical T helper 2	1.24 (range: 1.167 to 1.335)	0.06
		CD4+ regulatory T	1.333 (range: 1.225 to 1.52)	0.093
Fowler et al. [22]	Normal duodenum	CD4+ T-cells	1.413 (range: 1.121 to 1.755)	0.18
		CD8+ T-cells	1.86 (range: 1.423 to 2.828)	0.478
	Celiac disease duodenum	CD4+ T-cells	1.605 (range: 1.368 to 1.938)	0.196
		CD8+ T-cells	2.218 (range: 1.465 to 3.105)	0.478

**Table 4:** Summary of published studies providing data-permitting calculation of TCRbeta2/TCRbeta1 ratios.

Publication	Number and Diagnoses of Donors	Sample Type	Key Metrics Assessed	Results	Comments	Calculated TCRbeta2+/TCRbeta1+ Ratio (and Range)
Maciocia [17]	27 healthy donors	Peripheral blood mononuclear cells	Assessed TCRbeta1 against pan-TCRbeta	35% (range, 25–47%). MAITs and invariant natural killer T-cells contain a lower proportion of TCRbeta1+ cells.		1.86:1 (1.13–3:1)
Berg [32]	97 benign samples	Lymph node, tonsils, spleen, and bodily fluids	Assessed TCRbeta1 in subsets of CD3+ population.	CD4+: 43.79% (median) or 43.34% (mean) of CD3+ cells were TCRbeta1+ (range: 28.03–53.70; 95th percentile: 35.82–51.14). CD8+: 37.98% (mean) or 37.89% (median) of CD3+ cells were TCRbeta1+. (range: 15.46–59.89; 95th percentile: 26.64–50.52).	CD4+ cells: 95th percentile of 35.8–51.1% TCRbeta1+: CD8+ cells: 95th percentile of 36.5–50.8%. The study chose conservative cutoffs, consistent with Horna et al. [16]	CD4+ cells: 1.31:1 (range: 0.86:1–2.57:1) CD8+ cells: 1.63:1 (range: 0.67:1–5.47:1)
Ferrari [31]	4 healthy donors	Peripheral blood mononuclear cells	TCRbeta1 and TCRbeta2 dual staining	38:62 TCRbeta1/TCRbeta2		1.63:1
Horna [30]	24 healthy donors	Peripheral blood mononuclear cells	Assessed TCRbeta1 in subsets of CD3+ population.	Approximately 30–60% of CD4+ cells are TCRbeta1+ (JOVI1) across 4 subsets defined by CD7 and CD26 positivity/negativity		CD4: 0.67:1–2.33:1
Horna [16]	104 benign patient samples and 39 healthy donor samples	Peripheral blood mononuclear cells	TCRbeta1 and TCRbeta2 dual staining	Not possible to extract exact results.	Monotypia defined as >85% TCRbeta1+ or TCRbeta2+ cells	Range: 0.18:1–5.7:1
Waldron [33]	46 healthy donors	Peripheral blood mononuclear cells	Assessed TCRbeta1 against CD3.	CD4+ T-cells: 41% (30–48%) TCRbeta1+; CD8+ T-cells: 33% (22–49%) TCRbeta1+	TCRbeta monotypia defined as a TCRbeta1-negative population > 82% (CD4) or 88% (CD8), or a TCRbeta1-positive population > 68% (CD4) or 72% (CD8)	CD4: 1.44:1 (range: 1.08:1–2.33:1) CD8: 2.33:1 (range: 1.04:1–3.55:1)

**Table 5:** Analysis of TCRbeta1 and TCRbeta2 immunostaining of tissue sections containing T-cell lymphomas, with BaseScope^TM^ and Q-PCR corroboration. Ratios of immunohistochemical (IHC) and BaseScope^TM^ staining were derived from a consultant pathologist estimating the percentage of positive cells with each stain to the nearest 10%. Representative images are shown in Figure 5 and Figure 6 and Supplementary Figures S5 and S6.

Case Number	Site	Diagnosis	Biomed-2 Clonality Results	IHC Cell TCRbeta2/TCRbeta1	BaseScope^TM^ *TRBC2*/*TRBC1*	Q-PCR *TRBC2*/*TRBC1*	Comments
9	Lymph node	Peripheral T-cell lymphoma, NOS	Clonal TRB and TRG	<0.01:1 (TCRbeta1/TCRbeta2 > 100:1 (when assessing strong staining)). Tumor cells are strongly TCRbeta1+ (membranous distribution) with weak cytoplasmic TCRbeta2 staining.	<0.01:1 (*TRBC1*/*TRBC2* > 100:1 (when assessing strong staining)). Tumor cells contain approximately 10 times as many *TRBC1* as *TRBC2* transcripts.	0.66:1	Dual expression at the transcript and protein levels. Shown in Figure 5 and Supplementary Figure S5 panels E and F.
10	Lymph node	Peripheral T-cell lymphoma, NOS	Clonal TRB and TRG	10:1	97:1	10.54:1	
11	Lymph node	Peripheral T-cell lymphoma, NOS	Clonal TRB and TRG	>1000:1	>1000:1	458.34:1	Shown in Figure 5 and Supplementary Figure S5 panels C and D.
12	Skin (ear)	Indolent CD8+ T-cell lymphoma	Clonal TRB and TRG	>1000:1	>1000:1	2.17:1	Less *TRBC2*-skewed Q-PCR results than BaseScope^TM^ results are due to RNA extraction from peritumoral benign lymphocytes (no microdissection undertaken).
13	Skin (forehead)	CD4+ cutaneous T-cell lymphoma, unclassifiable	Clonal TRB and TRG	0.125:1 (TCRbeta1/TCRbeta2 = 8:1)	<0.25:1 (*TRBC1*/*TRBC2* 8:1.	0.74:1	Shown in Figure 6 and Supplementary Figure S6 panels A and B.
14	Skin (back)	Sézary syndrome	Clonal TRB and TRG	10:1 (but very low TCRbeta2 expression level)	5:1	4.16:1	
15	Skin (elbow)	Primary cutaneous anaplastic large cell lymphoma or transformed mycosis fungoides	Clonal TRB and TRG	50:1	>100:1 (when assessing strong staining). Each tumor cell contains approximately 5 times as many *TRBC2* as *TRBC1* transcripts.	6.05:1	Dual expression at the transcript, but not the protein level.
16	Skin (scrotum)	Transformed mycosis fungoides	Clonal TRB and TRG	0.05:1	0.01:1	0.07:1	Shown in Figure 5 and Supplementary Figure S5 panels A and B.
17	Skin (arm)	Transformed mycosis fungoides	Clonal TRB and TRG	>1000:1	>1000:1 (for counting cytoplasmic transcripts only, but all tumor cells also have high levels of aberrantly distributed nuclear *TRBC1* transcript.)	2.77:1	Shown in Figure 6 and Supplementary Figure S6 panels C and D.
18	Skin (back)	Mycosis fungoides	Clonal TRB and TRG	>100:1	99:1	2.81:1	
19	Vulva	CD30+ cutaneous T-cell lymphoma, unclassifiable	Clonal TRB and TRG	<0.001:1 (TCRbeta1/TCRbeta2 > 1000:1)	<0.001:1 (*TRBC1*/*TRBC2* > 1000:1), but all *TRBC1+* tumor cells co-express *TRBC2* transcripts with a *TRBC1*:*TRBC2* transcript ratio in each cell at around 3:1.	0.68:1	
20	Skin (back)	Cutaneous T-cell lymphoma, unclassifiable	Clonal TRB and TRG	6:1	10:1	1.35:1	
21	Skin (buttock)	CD8 positive cutaneous T-cell lymphoma, unclassifiable	Clonal TRB and TRG	>1000:1	>1000:1	10.63:1	Shown in Figure 6 and Supplementary Figure S6 panels E and F.

## Data Availability

All relevant datasets associated with this study are contained either in the main article or the Supplementary Materials.

## Supplementary Materials

The following supporting information can be downloaded at https://www.mdpi.com/article/10.3390/diagnostics14222479/s1. Figure S1: *TRBC1* (M12887.1) and *TRBC2* (M12888.1) 3′ untranslated region sequences, used for detection by BaseScope^TM^. Figure S2: Synthetic construct used as a PCR template in the pUCIDT (KanR) vector, with the insert comprising the 3′UTR of *TRBC1*, a 24 base random spacer containing an ECOR1 site, followed by the 3′UTR of *TRBC2*. Figure S3: Comparison of *TRBC1* and *TRBC2*-specific primer efficiency. Figure S4: BaseScope^TM^ corroboration of Figure 3. Figure S5: BaseScope^TM^ corroboration of Figure 5. Figure S6: BaseScope^TM^ corroboration of Figure 6. Table S1: RNASeq *TRBC1*/*TRBC2* ratio calculations.
