# Supplementary material for: Demonstration of T-Cell Monotypia Using Anti-TCRbeta1/2 (TRBC1/2) Immunostaining as a Rapid and Cost-Effective Alternative to PCR-Based Clonality Studies for the Diagnosis of T-Cell Lymphoma
Source: Diagnostics (Basel). 2024 Nov 6;14(22):2479. doi: 10.3390/diagnostics14222479 (PMC11593183; doi:10.3390/diagnostics14222479)
Supplement: Supplementary file 1 [file diagnostics-14-02479-s001.zip › Supplementary Figures combined FINAL.pdf]

GCCCTTGTGTTGATGGCCATGGTCAAGAGAAAGGATTTCTGAAGGCAGCCCTGGAAG

TGGAGTTAGGAGCTTCTAACCCGTCATGGTTTCAATACACATTCTTCTTTTGCCAGCGCT

TCTGAAGAGCTGCTCTCACCTCTCTGCATCCCAATAGATATCCCCCTATGTGCATGCACA

CCTGCACACTCACGGCTGAAATCTCCCTAACCCAGGGGGACCTTAGCATGCCTAAGTG

ACTAAACCGGTTAGGTA**GAATTC**GCGCGGTGGGCCCTCGTGCTGATGGCCATGGTCAA

GAGAAAGGATTCCAGAGGCTAGCTCCAAAACCATCCCAGGTCATTCTTCATCCTCACCC

AGGATTCTCCTGTACCTGCTCCCAATCTGTGTTCCTAAAAGTGATTCTCACTCTGCTTCTC

ATCTCCTACTTACATGAATACTTCTCTCTTTTTTCTGTTTCCCTGAAGATTGAGCTCCCAA

CCCCCAAGTACGAAATAGGCTAAACC

Supplementary Figure S1. Synthetic construct used as a PCR template in the pUCIDT (KanR) vector, with the insert comprising the 3’UTR of TRBC1, a 24 base random spacer containing an ECOR1 site, followed by the 3’UTR of TRBC2.

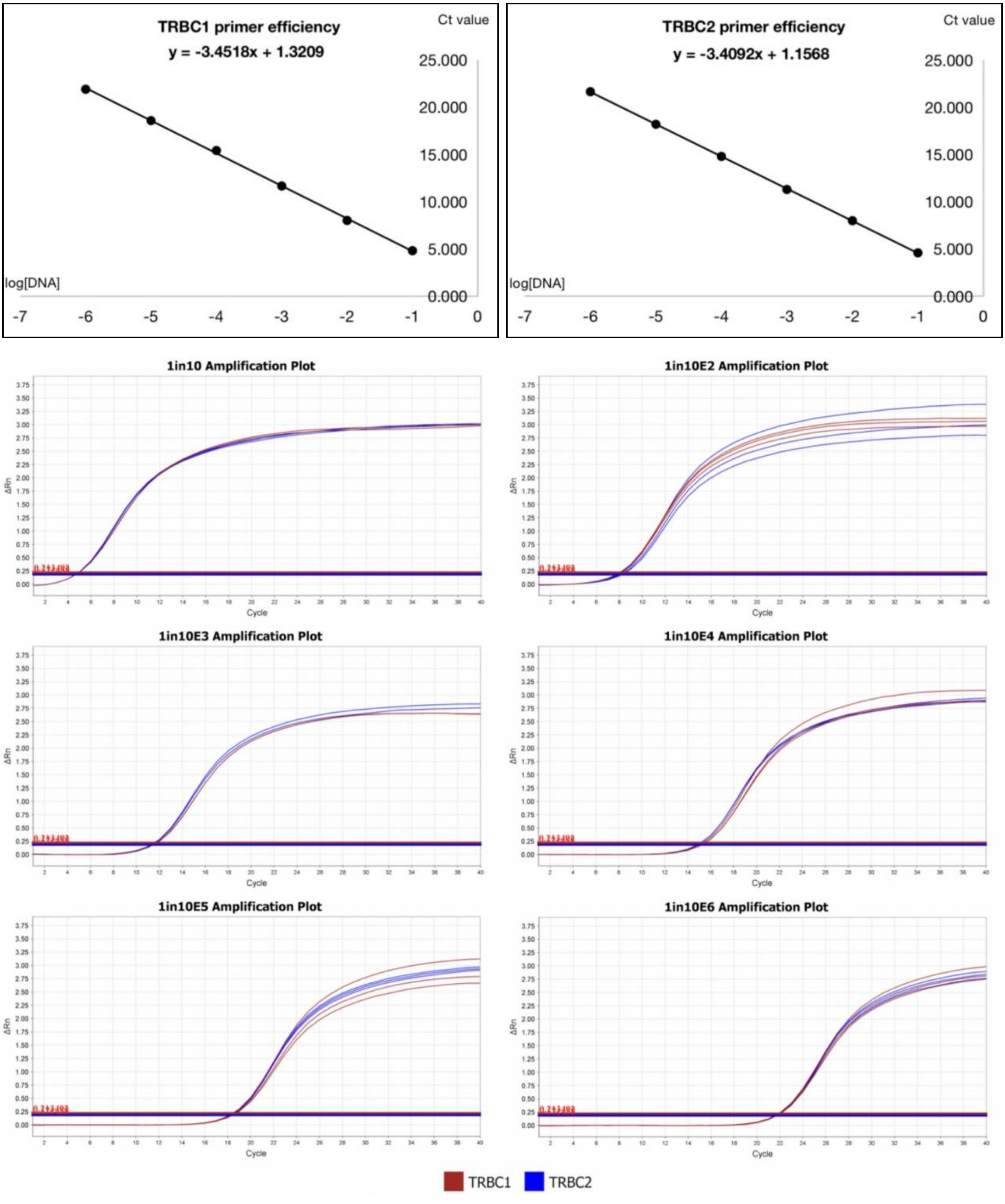

Supplementary Figure S2. Demonstration of equivalent TRBC1 and TRBC2-specific primer efficiency

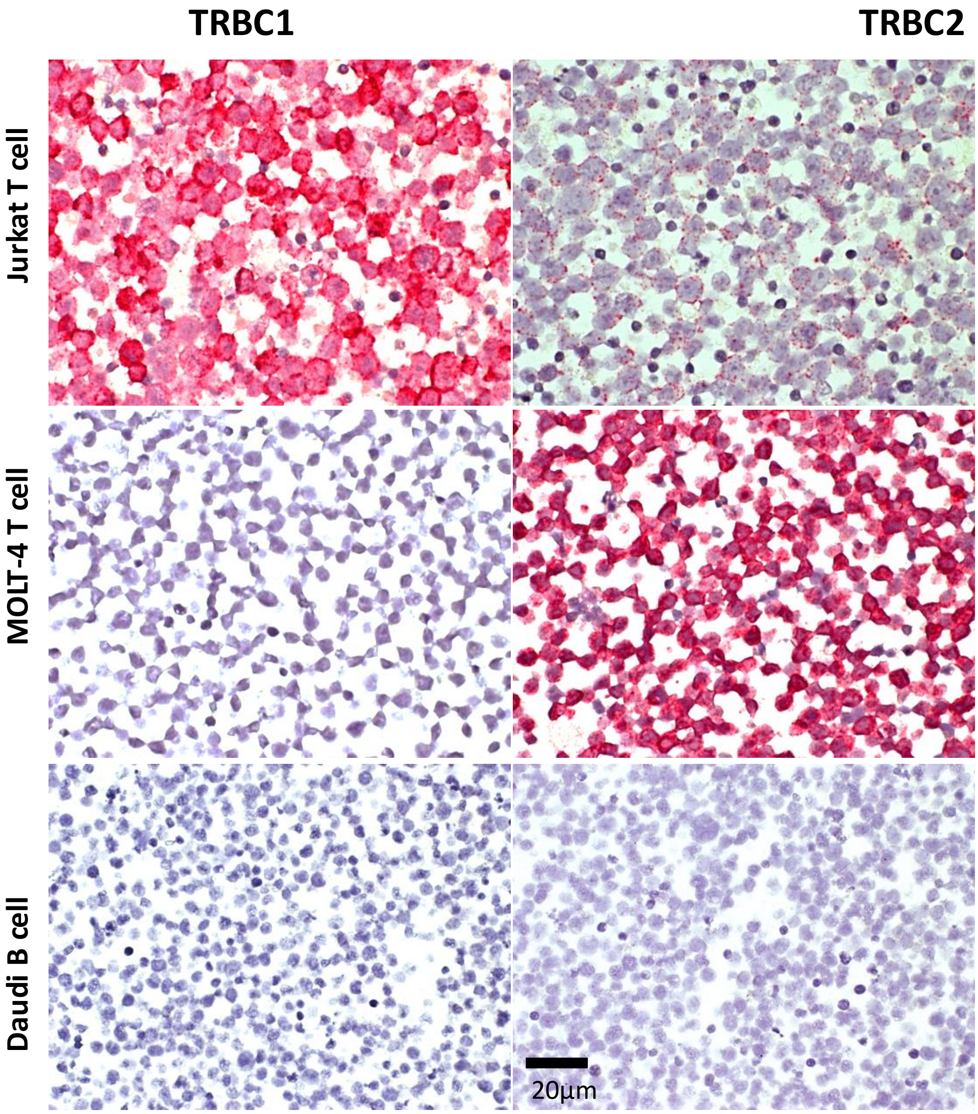

Supplementary Figure S3. BaseScope™ staining for TRBC1 and TRBC2 on key cell lines for which TCRbeta1 and TCRbeta2-specific immunostaining is shown in figure 3.

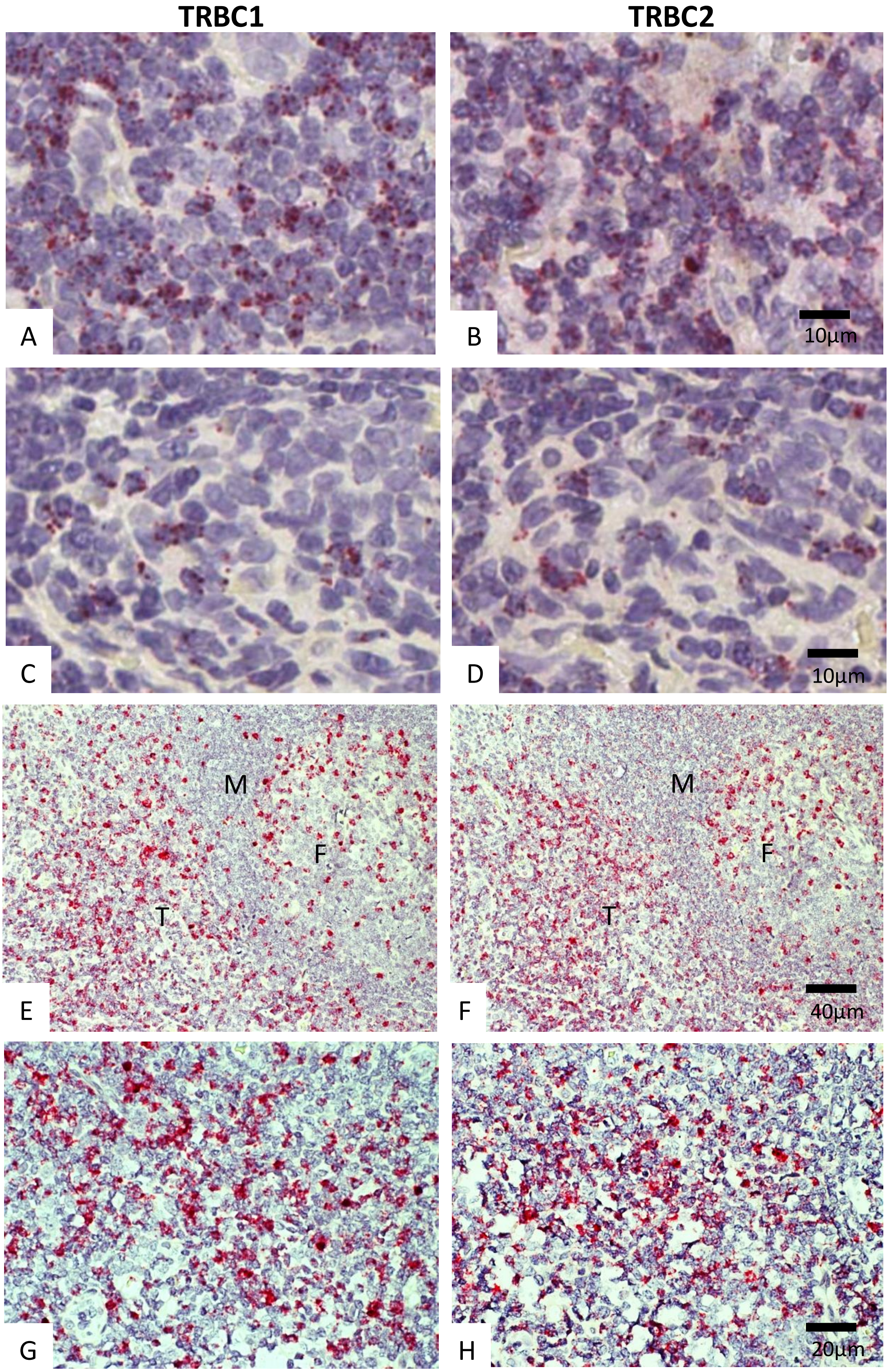

Supplementary Figure S4. BaseScope^{TM} staining for TRBC1 and TRBC2 on a benign lymph node paracortex (case 3; A and B) and secondary B-cell follicle, in which there are follicular helper T cells (C and D). BaseScope^{TM} staining of tonsil (case 1) shown on low magnification in E and F (F = follicle centre, M = mantle/ marginal zone, T = T-cell zone). High magnification of T-cell zone is shown in G and H.

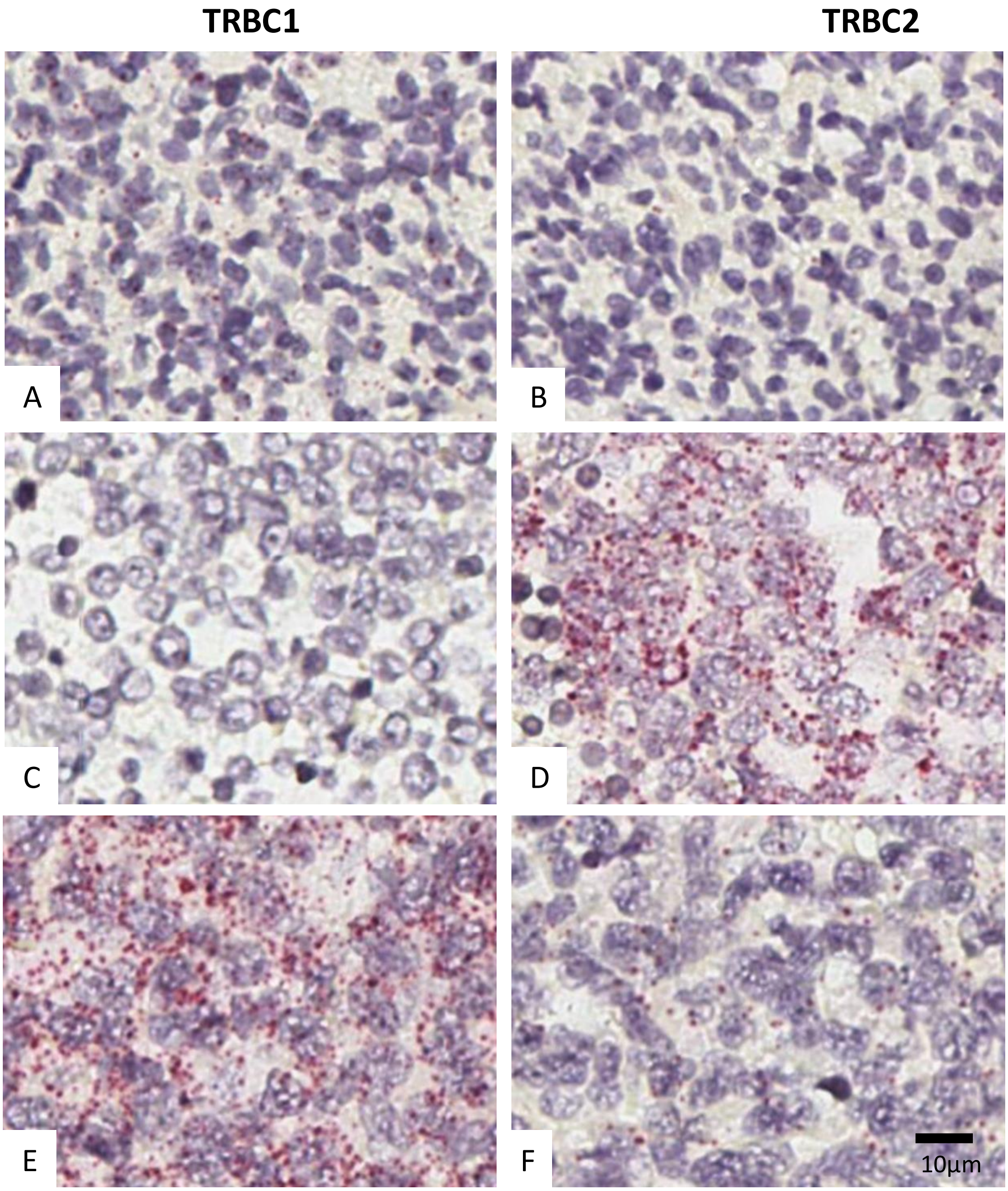

Supplementary Figure S5. Photomicrographs of BaseScope™ staining of FFPE sections for TRBC1 (left hand panels) and and TRBC2 (right hand panels). A and B. Cutaneous lymphoma (transformed mycosis fungoides) in scrotal skin (case 16) in A and B, showing limited RNA preservation, but clear TRBC1-restriction. C and D. Peripheral T-cell lymphoma, NOS, in a lymph node (case 11), showing clear TCRbeta2-restriction. E and F. Peripheral T-cell lymphoma, NOS, in a lymph node (case 9), showing predominentTRBC1 expression, with weaker TRBC2 co-expression. Scale bars is 10 microns and pertains to all panels. Corresponding immunohistochemical staining is shown in Figure 5 and Q-PCR results are presented in Table 5.

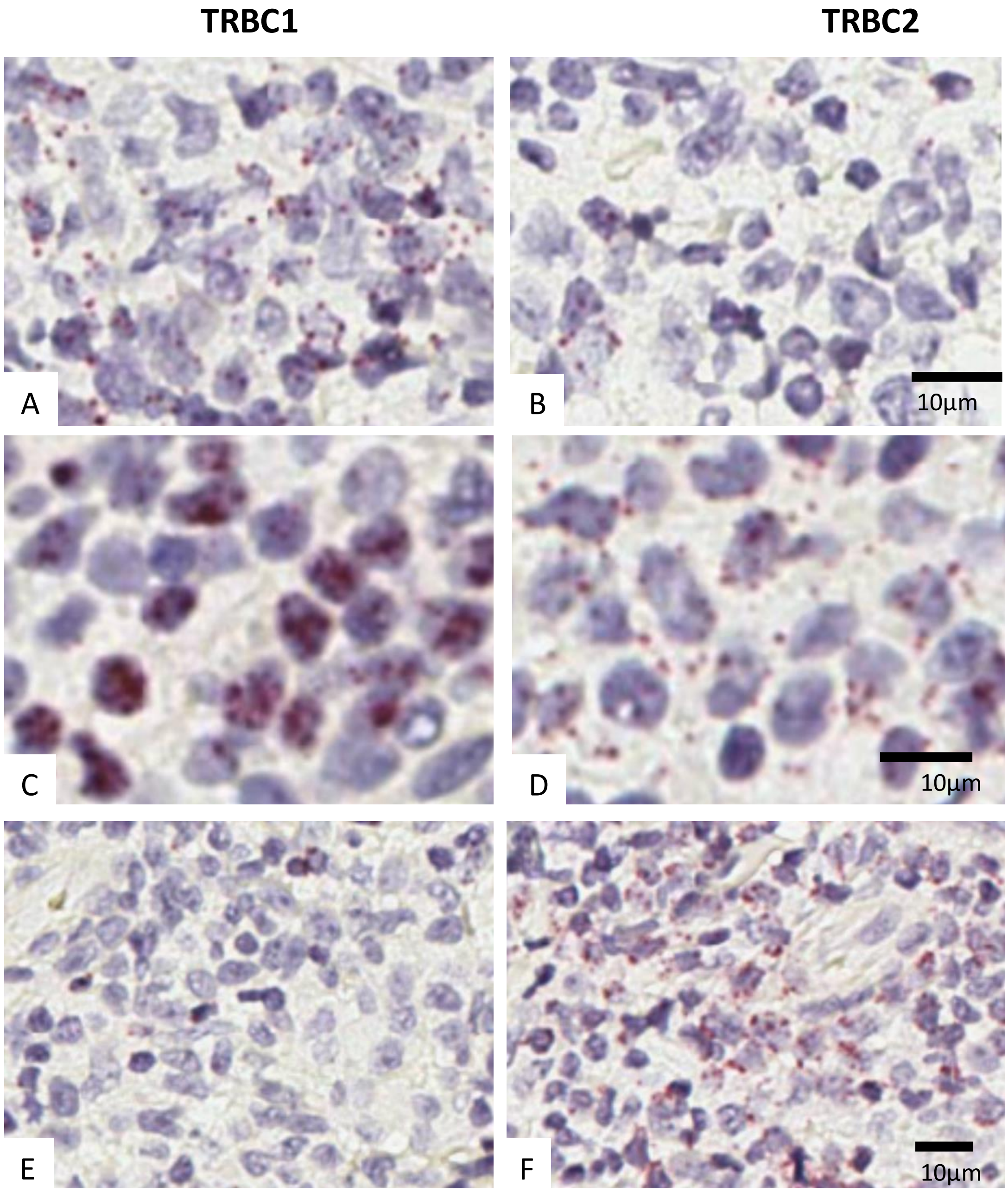

Supplementary Figure S6. Photomicrographs of BaseScope^{TM} staining of FFPE sections for TRBC1 (left hand panels) and and TRBC2 (right hand panels). A and B. Unclassifiable CD4+ cutaneous T-cell lymphoma (case 13) showing clear TRBC1-restriction. C and D. Cutaneous T cell lymphoma (transformed mycosis fungoides) (case 17), showing TCRbeta2 restriction, although at transcript level, there is cytoplasmic *TRBC2*, as seen in all the other cases examined, but very strong nuclear *TRBC1*. E and F. CD8 positive cutaneous T cell lymphoma, possibly acral lymphoma (case 21), showing clear TCRbeta2-restriction. Scale bars are 10 microns. Corresponding immunohistochemical staining is shown in Figure 6 and Q-PCR results are presented in Table 5.
